# Context-aware multi-token concept recognition of biological entities

**DOI:** 10.1186/s12859-021-04248-8

**Published:** 2021-10-21

**Authors:** Kwangmin Kim, Doheon Lee

**Affiliations:** 1 Department of Bio and Brain Engineering, Korea Advanced Institute of Science and Technology, Daejeon, South Korea; 2 Bio-Synergy Research Center, Daejeon, South Korea

**Keywords:** BERT, Concept recognition, Entity normalization, Gene ontology

## Abstract

**Background:**

Concept recognition is a term that corresponds to the two sequential steps of named entity recognition and named entity normalization, and plays an essential role in the field of bioinformatics. However, the conventional dictionary-based methods did not sufficiently addressed the variation of the concepts in actual use in literature, resulting in the particularly degraded performances in recognition of multi-token concepts.

**Results:**

In this paper, we propose a concept recognition method of multi-token biological entities using neural models combined with literature contexts. The key aspect of our method is utilizing the contextual information from the biological knowledge-bases for concept normalization, which is followed by named entity recognition procedure. The model showed improved performances over conventional methods, particularly for multi-token concepts with higher variations.

**Conclusions:**

We expect that our model can be utilized for effective concept recognition and variety of natural language processing tasks on bioinformatics.

## Background

As biological databases grow rapidly in size and natural language processing technology advances, the importance of automatic information extraction from texts is increasing more than ever. Among them, concept recognition in natural language texts is one of the core tasks for biological information mining.

**Fig. 1:**
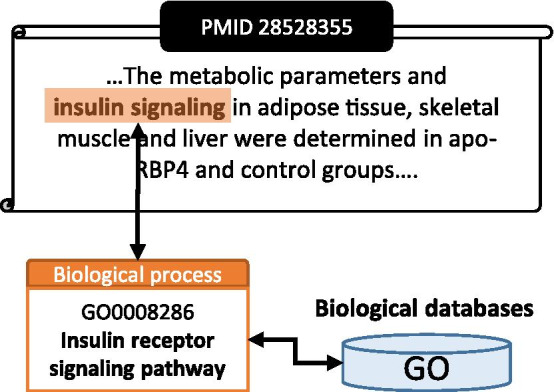
Definition of concept recognition and its linkage with biological databases

The feature characteristics of concept recognition task in bioinformatics is shown in Fig. 1. Concept recognition consists of two sequential stages: Named entity recognition (NER) and Named entity normalization (NEN). In NER, or text span detection stage, the location and the classified type of the entity mention in the given text is identified. NEN is also referred as various names, including entity disambiguation, entity linking or entity mapping. The common goal of this procedure is to identify a biological concept in the knowledgebase corresponding to the text span found in the NER stage and link it to an ontology identifier.

Concept recognition is considered to be a particularly important topic in bioinformatics since there are biological databases constructed for researchers. While there are vast amount of knowledge accumulated in the biological knowledge-bases, this knowledge cannot be utilized by performing NER alone. Even if a certain span of text is recognized as biological entities through NER, as shown in Fig. 1, the text span (“Insulin signaling”) should be 'linked' or 'mapped' into specific entity of the biological knowledgebase to derive meaningful biological information from text. To be more specific, to integrate recognized text spans and biological databases, the connection between the text spans and specific entities within the database should be identified as a form of certain ID (“GO0008286”). This demonstrates the biological importance of biological concept recognition.

**Fig. 2:**
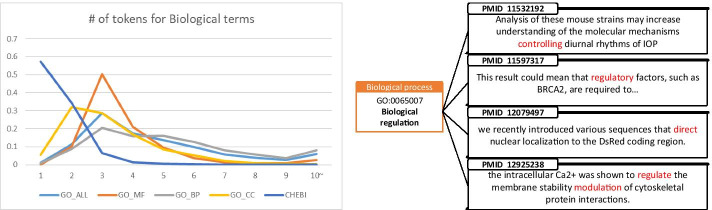
(Left) Distribution of term lengths in biological knowledgebase by concept types. (Right) Biological concepts with longer term length tend to have more variations in actual usage in text

Despite the importance of the concept recognition task, the performance of conventional methods so far has been open to improvement. In particular, the performance of the conventional methods was significantly degraded in the recognition of the multi-token concepts. Figure 2 (left) shows the distribution of terms lengths of the biological concepts collected from biological knowledge-bases [1]. It is noticeable that the length of the gene ontology terms tends to be longer, and these multi-token terms are known to be difficult to be recognized due to various factors.

The biggest factor that hinders the recognition of multi-token concepts is name variation. When multi-token concepts actually appear in the literature texts, the expressions of the concepts may be highly variant. As the number of word tokens constituting the biological concept increases, its variation also rapidly diversifies. Figure 2 (right) shows how variant a multi-token concept can be when used in actual literature text. These examples were selected from the 34 different textual expressions of a single biological concept “biological regulation”, collected from CRAFT corpus composed of only 97 literature texts. Some studies apply explicit solutions, such as using synonyms lists, but their effectiveness is largely limited. For example, Drugbank [2] contains four synonyms for the drug concept “Aspirin”, which is sufficient to cover most cases where Aspirin is mentioned in the actual text. However Gene Ontology [1] contains six synonyms for the biological process concept “positive regulation of biological process”, which is clearly insufficient to reflect the extensive potential variation of the term. Due to this factor, conventional dictionary-based concept recognition methods showed insufficient performances on concepts with relatively longer, multi-token terms [3, 4].

It is known that biological knowledge-bases, such as Gene ontology, contain a variety of semantic information ranging from definition text of the concepts, neighboring concepts and corresponding hierarchical relations among concepts. In this research, we have extracted the contextual information from the biological knowledge-bases to infer the improved normalization for the biological concepts.

## Previous work

After the first introduction of Bidirectional Encoder Representations from Transformers (BERT) in 2018, it has recorded state-of-the-art results in a variety of natural language processing tasks. The key feature of BERT is application of bidirectional attention-based transformer. The BERT model is trained on two tasks: first is the masked language task, which use the output of the masked word’s position to predict the masked word. The other is the next sentence prediction task, which predict likelihood of a sentence belongs after another sentence. The adoption of the BERT-based models has become even more promising option for biological NLP tasks, since the BERT-based models specifically pre-trained for biological domain has been introduced [5, 6]. Those pre-trained models are known to even outperform BERT in various biological NLP tasks [7].

There has been a general BERT-based workflow established for NER task which is being widely used. However, there isn't an established BERT model for entity normalization. This is due to the nature of the BERT language model, where the vector value of the word piece token is determined by the context of the surrounding, making it difficult to derive the vector of every entity mention in the text in advance.

There are several studies that have addressed this issue and performed concept recognition task utilizing BERT language model. Hailu et al. [8] approached concept normalization as a sequence-to-sequence mapping task, normalizing characters in the text spans to the sequence of characters that constitute and ontology identifier, considering the normalization task as a kind of language translation. The biological plausibility of the model, however, is questionable. Unlike language translation where sequence-sequence model is frequently adopted, each characters of an ontology identifiers are semantically related each other. Moreover, the similarities among text span sequences are not necessarily preserved in among ontology identifiers, and vice versa. Thus, semantically similar biological concepts cannot be assumed to have similar identifiers, and the biological concepts with similar identifiers cannot be assumed to have similar meanings. Following three gene ontology concepts well depicts the issue: *GO: 0008285*—*Negative regulation of cell proliferation*. *GO:0008286*—*Insulin receptor signaling pathway*. *GO:0032869*—*Cellular response to insulin stimulus*.

Recently, an ‘end-to-end’ normalization approach has been proposed to tackle the issue. In this approach, the model does not train NER and NEN as separate sequential steps. Instead, it is trained as a single joint task. Since errors in the previous steps are not cascaded to the next step, the joint model showed significant improvement in performance [9]. Furrer et al. [10] also applied similar approach in biomedical domain for concept recognition, and obtained promising performances. However, the method basically considers the normalization process as multi-class classification task. That is, the model cannot label the ‘unseen concepts that were not appeared in the training set. This limitation reduces the advantage of the model to be used for general tasks for broader dataset. The method utilize a separate dictionary-based normalization method to cover the part of the dataset [11]. But the performance on the unseen concepts were at the level of a typical dictionary-based methods, especially in the multi-token concepts.

While there have been various approaches for biological concept recognition, there have not been many approaches that utilized contextual information of the ontology. In this study, we have developed a concept recognition methods utilizing these various contextual information that can be accessed from the ontology structure.

## Methods

The overall procedure of the study can be summarized as follows. First, we have adopted NER module based on bioBERT language model to identify the text spans of the mentions according to known biological concepts from the input text. Next, we have derived the vector representations of the obtained concept mentions with given text mentions, and concept names and definitions texts of the candidate biological concepts. Using the cosine similarities among vector represented concept mentions, concept names and concept definitions, the normalized concept was predicted. Detailed explanation about each step will be given in the sections below.

### Named entity recognition (NER)

**Fig. 3:**
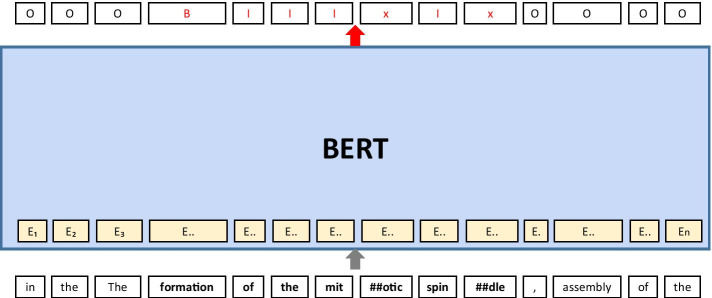
Named entity recognition process based on BERT model

We have utilized the NER module provided by bioBERT model for NER step. BioBERT [5] is a domain-specific BERT model pre-trained on biomedical corpora, and it has been identified that bioBERT outperforms BERT in domain-specific tasks. Figure 3 shows the named entity recognition process based on bioBERT model. Input text is tokenized into Wordpiece tokens [12], then fed into pre-trained bioBERT model. The model is fine-tuned to predict the label corresponding to each tokens. In the NER task, each tokens were trained to be tagged as one of the three labels to represent the text span of the named entities: B (Begin), I (Inside), O (Outside) of the text span.

### Concept normalization

For Concept normalization, we have derived vector representations of each text spans of the mentions that were obtained in the NER stage, concept titles and concept definitions of knowledgebase.

#### Structure of ontology concepts

The ontologies used in the study follows the open biomedical ontologies (OBO) format [13]. An example case of ontology concept is described in the following paragraph.

- **id:** GO:0007596
- **name:** blood coagulation
- **def:** "The sequential process in which the multiple coagulation factors of the blood interact, ultimately resulting in the formation of an insoluble fibrin clot; it may be divided into three stages: stage 1, the formation of intrinsic and extrinsic prothrombin converting principle; stage 2, the formation of thrombin; stage 3, the formation of stable fibrin polymers."
- **synonym:** "blood clotting" EXACT
- **is_a:** GO:0007599 ! hemostasis
- **is_a:** GO:0050817 ! coagulation
- **relationship: part_of** GO:0042060 ! wound healing

**Fig. 4:**
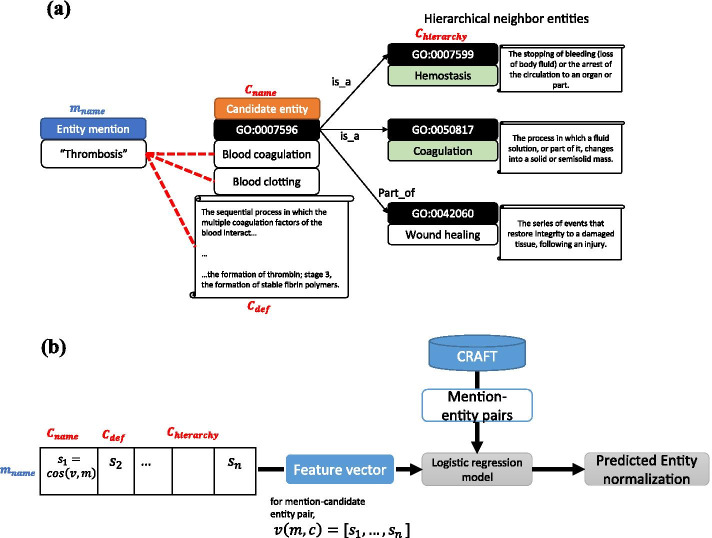
Concept normalization procedure. **a** An entity mention and corresponding contexts. **b** Model for entity normalization prediction

These information given for each ontology concepts is a valuable source of context that can be utilized for normalization. Figure 4a illustrates the relations among a detected entity mention $m_{{name}}$ and candidate concepts that can be normalized to the mention. For each candidate concept, we can extract the concept name $C_{{name}}$, concept definition $C_{{def}}$, hierarchical context $C_{{hierarchy}}$.

From the ontology, the name, definition, synonyms, hierarchical relations (is_a, part_of) were extracted for vector representation. Each synonyms were regarded as individual concepts with a shared common definition, while the name of the concepts have difference values that are distinguished each other. Among various types of relations described in GO ontology, we have selected *is_a* and *part_of* relations as those relations occupy majority of the ontology and conveys the hierarchical relation we intend to extract.

#### Vector representation of mentions and concepts

We have adopted Sentence-BERT [14], a BERT-based model fine-tuned for semantically meaningful sentence embedding. While BERT base models show better performance than the conventional language model, the vectors of each word tokens are not fixed on a particular value. As a result, the base BERT model is not capable of predicting vectors of untrained sentence queries. The main advantage of utilizing the Sentence-BERT model is that vector representations based on the BERT model can also be derived for input query text given without additional fine-tuning. The Sentence-BERT model is built on the Siamese network and is trained to predict the similarity score between two input sentence vectors. That is, the model is trained to output high similarity scores for similar sentences. As a result, the Siamese network model is able to predict a representation vector corresponding to the input sentences through metric learning, which can be used to calculate similarity between any arbitrary sentence pairs.

As a result, vector representations for the extracted mention, concept names, concept definitions, and hierarchical contexts were derived.

#### Normalization model

Figure 4b shows the normalization model we have constructed. First, for the concept mention obtained in the previous step and for each candidate concepts, cosine similarities of each mention-context pairs are calculated. Then the cosine similarities between mention-name, mention-definition and mention-hierarchy pairs were concatenated to form a feature vector. In the case of hierarchical context, up to 3 hierarchical contexts were reflected.to construct the feature vector. Finally, we constructed a regression model using feature vectors as input data and mention-concept pair extracted from CRAFT corpus as golden standard set.

### Experimental materials

To train and test the model, The Colorado Richly Annotated Full-text (CRAFT) corpus was used [15]. The corpus consists of 97 full-length biomedical journal articles, and the articles are manually annotated with 10 distinct biomedical ontologies [16]. For the research, we have focused on biological process entities in gene ontology (GO_BP) as they have the longest term length on average, thus having the highest variations on their actual usage patterns.

### Evaluation procedure

The official evaluation suite provided by CRAFT shared task was used for the evaluation. The suite measures performance in terms of two distinct measures: Slot Error Rate (SER) and F-score (F1). Both measures are calculated considering the counts of matches, insertions, deletions and substitutions between the predicted sequences. SER is defined as the ratio of the total numbers of slot errors, including substitutions, deletions and insertions divided by the total number of slots in the reference [17]. Alternatively, it can be represented as:

$$SER = \frac{{S + D + I}}{{C + S + D}} = \frac{{Total\;number\;of\;slot\;error}}{{Total\;number\;of\;slots\;in\;reference}}$$

where S, D, I, C each corresponds to the number of substitutions, deletions, insertions and correct slots. In term of F1 score, only the case of exact span match was evaluated as positive answers.

## Results

The experimental result of proposed concept recognition model is shown in Table 1. For concept recognition of GO_biological process, our proposed model showed improved performance compared to the conventional models, in terms of both SER and F1 scores. In particular, it largely outperformed the dictionary-based method [3] which again shows that performance of the dictionary-based approaches is significantly lowered in the multi-token concept recognition task.

**Table 1:** Concept normalization performance on GO_biological process concepts

Model	**SER**	**F1 score**
(Funk, 2014) [3]	–	0.33
(Hailu, 2019) [8]	0.56	0.64
Context + Cosine similarity	0.57	0.616
Context + Regression	**0.53**	**0.654**
(Furrer, 2019) [10] (trained concepts)	0.30	0.80
(Furrer, 2019) (unseen concepts)		0.22

Best result for each measure is indicated in bold-face

In addition, the performance evaluation of concept recognition in the Furrer's end-to-end method is also indicated. In the evaluation of the CRAFT corpus, this method surpassed all other methods. However, as previously explained, the method is expected to show competitive performance only in limited datasets as it can only recognize concepts that have been used for learning. For unseen concepts that were not used for learning, this method show relatively poor performance.

Table 2 shows a case example how the contextual information contributes to correct normalization of mentions into concepts. The Mention “Formation of the mitotic spindle” is recognized in the NER stage, and normalized into the concept  *"GO:0090307—Mitotic spindle assembly"*. The cosine similarity among the mention, concept names and definitions are shown. For the cosine similarity among vectors derived from concept names, the concept “*GO:0000132—Orienting of mitotic spindle”* shows the highest similarity with the mention, which is a natural result considering that it has the most number of identical tokens. However, in terms of definition similarity, the most similar concept is “*GO:0090307—Mitotic spindle assembly”.* The definition text of it contains the words that are semantically similar to ‘formation; and also the overall meaning of the text is closely related. Considering the overall definitions of the concepts, it is evident that “*GO:0090307*—*Mitotic spindle assembly”* is a proper normalization for the mentions, which was cross-checked via golden standard set of CRAFT corpus.

**Table 2:** Concept name candidates for mention *“Formation of the mitotic spindle”*

Identifier	Concept name	Definition	Concept name similarity	Definition similarity
GO:0090307	Mitotic spindle assembly	The aggregation, arrangement and bonding together of a set of components to form the spindle that contributes to the process of mitosis	0.9281	**0.815**
GO:0000132	Orienting of mitotic spindle	A cell cycle process that sets the alignment of mitotic spindle relative to other cellular structures	**0.9305**	0.7417
GO:1901673	Regulation of mitotic spindle assembly	Any process that modulates the frequency, rate or extent of mitotic spindle assembly	0.9156	0.7432

Best results for each similarity measure is indicated in bold-face

## Discussion

Our model focuses on the recognition of gene ontology entities, especially on biological process entities. However, it can be easily extended to deal with other types of biological entities, when given proper context knowledge-bases. Thus, our model can be utilized to build and maintain biological databases with extracted biological concepts from the literature.

Although not covered in detail, it is also a notable feature of our model that normalized terms corresponding to a given entity can always be found through the features based on cosine similarity. On the other hand, classical rule-based models often fail to find a normalized term for a particular mention. This is a trade-off between sensitivity and specificity, and does not necessarily mean the superiority of our model. However, considering the intrinsic purpose of the entity normalization task, mapping 'semantically similar' term can be significantly considered for a particular purpose, even though the normalized term does not exactly match the gold standard set.

Our model, however, has some limitations to be noted. The advantages of our model are relatively reduced when the length of the entity term is short and the term variation is limited. In this case, conventional rule-based normalization models applied with synonym dictionaries would demonstrate competitive performance. Ultimately, we expect that applying a differentiated entity normalization model based on the characteristics of the entity type would be a promising approach.

## Conclusions

This paper presents a novel methods in biological concept recognition which utilizes the contextual information of the concept knowledgebase. The key idea of the model is collecting the extensive context information for each ontology concepts from knowledgebase, and deriving the sentence vectors for each entities using the Siamese network model. The model showed a meaningful performance improvement compared to the conventional methods, and its advantage was more evident in the recognition task of multi-token concepts.

This implies that the model can be more promising in certain domains where conventional concept recognition methods has been ineffective. Also, the core idea of the model, utilizing the information collected from biological databases, can be extended to cover more variety of biological information.

## Data Availability

The datasets generated and analyzed during the current study are available in the GitHub repository, https://github.com/kmkim2/CAMCOR.

## Abbreviations

BERT: Bidirectional encoder representations from transformers
CRAFT: Colorado richly annotated full text
GO: Gene ontology
GO_BP: Gene ontology biological process
NEN: Named entity normalization
NER: Named entity recognition
OBO: Open biomedical ontologies
SER: Slot error rate

## References

[1] Ashburner M, Ball CA, Blake JA, Botstein D, Butler H, Cherry JM (2000). Gene ontology: tool for the unification of biology. Nature.

[2] Wishart DS, Feunang YD, Guo AC, Lo EJ, Marcu A, Grant JR (2018). DrugBank 5.0: a major update to the DrugBank database for 2018. Nucl Acids Res.

[3] Funk C, Baumgartner W, Garcia B, Roeder C, Bada M, Cohen KB (2014). Large-scale biomedical concept recognition: an evaluation of current automatic annotators and their parameters. BMC Bioinform.

[4] Leaman R, Lu ZJB (2016). TaggerOne: joint named entity recognition and normalization with semi-Markov Models. Bioinformatics.

[5] Lee J, Yoon W, Kim S, Kim D, Kim S, So CH (2020). BioBERT: a pre-trained biomedical language representation model for biomedical text mining. Bioinformatics.

[6] Huang K, Altosaar J, Ranganath R. Clinicalbert: modeling clinical notes and predicting hospital readmission. 2019.

[7] Ji Z, Wei Q, Xu H (2020). Bert-based ranking for biomedical entity normalization. AMIA Summits Transl Sci Proc.

[8] Hailu ND, Bada M, Hadgu AT, Hunter LE (2019). Biomedical concept recognition using deep neural sequence models. bioRxiv.

[9] Broscheit S. Investigating entity knowledge in BERT with simple neural end-to-end entity linking. 2020.

[10] Furrer L, Cornelius J, Rinaldi F, editors. UZH@ CRAFT-ST: a Sequence-labeling approach to concept recognition. In: Proceedings of the 5th workshop on BioNLP open shared tasks; 2019.

[11] Furrer L, Jancso A, Colic N, Rinaldi F (2019). OGER++: hybrid multi-type entity recognition. J Cheminform..

[12] Wu Y, Schuster M, Chen Z, Le QV, Norouzi M, Macherey W, et al. Google's neural machine translation system: bridging the gap between human and machine translation. 2016.

[13] Smith B, Ashburner M, Rosse C, Bard J, Bug W, Ceusters W (2007). The OBO foundry: coordinated evolution of ontologies to support biomedical data integration. Nat Biotechnol.

[14] Reimers N, Gurevych I. Sentence-bert: sentence embeddings using siamese bert-networks. 2019.

[15] Cohen KB, Verspoor K, Fort K, Funk C, Bada M, Palmer M (2017). The colorado richly annotated full text (craft) corpus: multi-model annotation in the biomedical domain. Handbook of Linguistic annotation.

[16] Bada M, Eckert M, Evans D, Garcia K, Shipley K, Sitnikov D (2012). Concept annotation in the CRAFT corpus. BMC Bioinform.

[17] Makhoul J, Kubala F, Schwartz R, Weischedel R, editors. Performance measures for information extraction. In: Proceedings of DARPA broadcast news workshop; 1999: Herndon, VA.

